# The Extent of Engagement With Telehealth Approaches by Patients With Advanced Cancer: Systematic Review

**DOI:** 10.2196/33355

**Published:** 2022-02-17

**Authors:** William Goodman, Anne-Marie Bagnall, Laura Ashley, Desiree Azizoddin, Felix Muehlensiepen, David Blum, Michael I Bennett, Matthew Allsop

**Affiliations:** 1 Leeds Institute of Health Sciences University of Leeds Leeds United Kingdom; 2 School of Health Leeds Beckett University Leeds United Kingdom; 3 Leeds School of Social Sciences Leeds Beckett University Leeds United Kingdom; 4 Department of Emergency Medicine Brigham and Women's Hospital Boston, MA United States; 5 Department of Psychosocial Oncology and Palliative Care Dana-Farber Cancer Institute Boston, MA United States; 6 Brandenburg Medical School Theodor Fontane Neuruppin Germany; 7 Competence Center Palliative Care University Hospital Zurich Zurich Switzerland

**Keywords:** systematic review, advanced cancer, engagement, digital health, telehealth, mobile phone

## Abstract

**Background:**

Telehealth approaches are increasingly being used to support patients with advanced diseases, including cancer. Evidence suggests that telehealth is acceptable to most patients; however, the extent of and factors influencing patient engagement remain unclear.

**Objective:**

The aim of this review is to characterize the extent of engagement with telehealth interventions in patients with advanced, incurable cancer reported in the international literature.

**Methods:**

This systematic review was registered with PROSPERO (International Prospective Register of Systematic Reviews) and is reported in line with PRISMA (Preferred Reporting Items for Systematic Reviews and Meta-Analyses) 2020 guidelines. A comprehensive search of databases was undertaken for telehealth interventions (communication between a patient with advanced cancer and their health professional via telehealth technologies), including MEDLINE, Embase, CINAHL, PsycINFO, Cochrane Library, Sociological Abstracts, and Web of Science, from the inception of each electronic database up until December 31, 2020. A narrative synthesis was conducted to outline the design, population, and context of the studies. A conceptual framework of digital engagement comprising quantitative behavioral measures (frequency, amount, duration, and depth of use) framed the analysis of engagement with telehealth approaches. Frequency data were transformed to a percentage (actual patient engagement as a proportion of intended engagement), and the interventions were characterized by intensity (high, medium, and low intended engagement) and mode of delivery for standardized comparisons across studies.

**Results:**

Of the 19,676 identified papers, 40 (0.2%) papers covering 39 different studies were eligible for inclusion, dominated by US studies (22/39, 56%), with most being research studies (26/39, 67%). The most commonly reported measure of engagement was frequency (36/39, 92%), with substantial heterogeneity in the way in which it was measured. A standardized percentage of actual patient engagement was derived from 17 studies (17/39, 44%; n=1255), ranging from 51% to 100% with a weighted average of 75.4% (SD 15.8%). A directly proportional relationship was found between intervention intensity and actual patient engagement. Higher engagement occurred when a tablet, computer, or smartphone app was the mode of delivery.

**Conclusions:**

Understanding engagement for people with advanced cancer can guide the development of telehealth approaches from their design to monitoring as part of routine care. With increasing telehealth use, the development of meaningful and context- and condition-appropriate measures of telehealth engagement is needed to address the current heterogeneity in reporting while improving the understanding of optimal implementation of telehealth for oncology and palliative care.

**Trial Registration:**

PROSPERO (International Prospective Register of Systematic Reviews) CRD42018117232; https://www.crd.york.ac.uk/prospero/display_record.php?ID=CRD42018117232

## Introduction

### Background

Cancer ranks as a leading cause of death worldwide and is a leading cause of premature death in most countries [1]. For people living with advanced cancer, fluctuating unmet needs can be experienced over time with disease progression [2]. Common symptoms include pain, experienced in approximately two-thirds (66.4%) of patients with advanced disease [3], alongside breathlessness, nausea and vomiting, and fatigue [4]. Typically, individuals experience more than one symptom, with an average of 14 symptoms for those with advanced cancer [5]. Such physical symptoms often exist alongside deterioration across physical, psychological, social, spiritual, and overall quality of life (QOL) trajectories [6]. There remain gaps in supporting care delivery for patients with cancer, including barriers in health communication with health care providers, lack of care coordination, and challenges in accessing care [7].

Telehealth and telehealth interventions refer to a method in which the patient and health care professional can communicate clinical information remotely via a number of different mediums such as telephone, web-based methods, and mobile apps [8]. This method is increasingly used to deliver cancer care as it provides opportunities for efficient and flexible service delivery and enables clinicians to maintain involvement independent of the physical location of the patients or clinicians [9-12]. These characteristics have also driven their increased application to support delivery of care during the COVID-19 pandemic, enabling avoidance of direct physical contact while contributing to provision of continuous care in the community. Telephone-based approaches have been highlighted as a possible means of overcoming gaps in service delivery for patients with cancer [7], including reducing the travel required to access support services that can lead to physical, psychological, and financial stress [13,14]. Examination of telehealth approaches for patients with chronic diseases has found varying effects, with improved self-management of diabetes and reduced mortality and hospital admissions in heart failure, but these improvements have not been observed across other conditions, including cancer [8]. Emerging evidence is mixed, with a recent review that focused on all cancer stages demonstrating clinical equipoise, with no discernible difference between telehealth and usual care in improving QOL [15]. However, a recent systematic review focusing specifically on patients with advanced cancer and diverse web and technological interventions (largely providing psychosocial, self-management, and expert-guided support) found that most approaches suggested some degree of efficacy relating to QOL and psychosocial well-being [16]. However, we do not know how well people with advanced cancer engage with these interventions.

With emerging clinical validation demonstrating the potential of digital technology approaches to improve care and outcomes of patients with advanced cancer, usability must also be considered [17]. Subjective aspects of usability require a better understanding, specifically regarding user satisfaction and engagement [17]. Patient engagement can be an important factor in the success of health interventions, leading to better intended health outcomes for the patient and lower health care costs [18]. As such, the effectiveness of telehealth interventions in improving health outcomes is heavily dependent on patient engagement. However, patient engagement is a broad term that can cover multiple levels of how a patient interacts with an intervention. For the purposes of this review, with a focus on technology-based interventions, *engagement* will be used to refer to the specific quantitative measures of behavior of engagement as defined by Perski et al [19] (ie, comprising the frequency, amount, duration, depth of use, and other measures of use and interaction with a digital health intervention). A previous systematic review found that information technology platforms (eg, mobile phone devices, internet-based interventions, social media, and other web-based communication tools) can help engage patients in health care processes and motivate health behavior change [20]. However, interventions with the intention to help support patients in managing chronic conditions can be complex. There is a need to understand whether different aspects of telehealth interventions uniquely influence patient engagement, especially for patients with advanced cancer who often experience a high symptom burden and functional impairment [21]. Understanding patients’ engagement with telehealth interventions is necessary to further evaluate and refine the implementation of these emerging and promising approaches for patients with advanced cancer. Therefore, there is a need to understand how patients with advanced cancer engage with telehealth interventions and which aspects of these interventions may influence engagement.

### Objectives

Past systematic reviews have sought to synthesize the evidence of telehealth interventions among patients with cancer and survivors but have not explored interventions solely intended for and tested on patients with advanced, incurable cancer [15,16]. Understanding patient engagement can help us evaluate and refine further design, development, and evaluation of telehealth approaches for people with advanced cancer. A companion review [22] explored the clinical and cost-effectiveness of the interventions on health and health system outcomes, whereas this review synthesizes the data on patient engagement with the interventions. The aims of this review are as follows: (1) to characterize the extent of behavioral engagement of people with advanced, incurable cancer with telehealth interventions and (2) to explore factors that influence engagement with telehealth interventions.

## Methods

### Information Sources

This systematic review was registered with PROSPERO (International Prospective Register of Systematic Reviews; CRD42018117232). A systematic review of the literature was conducted in the following databases: MEDLINE, Embase, CINAHL, PsycINFO, Cochrane Library, Web of Science, and Sociological Abstracts, with studies included from the inception of each electronic database up until December 31, 2020. No lower cutoff date was chosen as there has not been a previous review looking into engagement with telehealth interventions in this population. An example search strategy used for MEDLINE can be found in Multimedia Appendix 1 and includes keywords and medical subject headings. The development of the search strategy was supported by information specialists at the University of Leeds. This search was supplemented by forward and backward citation searching of key papers. This review was reported in line with the PRISMA (Preferred Reporting Items for Systematic Reviews and Meta-Analyses) 2020 guidelines. The Centre for Reviews and Dissemination guidelines directed our process for conducting this systematic review and the decisions made [23].

### Eligibility Criteria

Studies were eligible for inclusion in the review if the following applied:

1. They involved a telehealth intervention, which is defined as “any intervention in which clinical information is transferred remotely between patient and health care provider, regardless of the technology used to record or transmit the information” [8]. This could include symptom measuring or monitoring (eg, Patient-Reported Outcome Measures); education, information giving, and support, including decision aids and advanced care planning; psychological interventions; or medical consultation (telemedicine or teleconsultation). Participants could be located anywhere as long as the intervention that was carried out conformed to the telehealth definition.
2. They included participants of any age who were living with cancer of any type that could not be cured (advanced, metastatic, or terminal). This included people who had been treated with curative intent but whose cancer had recurred or progressed, those not being treated with curative intent, and those at or near end of life.
3. They included a measure of engagement as an outcome or reported as part of the study findings. In this review, we used the measures conceptualized as behavior that were identified by Perski et al [19]: frequency, amount, duration, and depth of use.
4. The studies were carried out in any country at any time.
5. Risk of bias was not used as a selection criterion for inclusion in the review.

Studies were excluded when the following applied:

1. The participants included patients with cancer currently being treated with curative intent, and the studies had mixed populations (ie, not 100% of the sample were people with cancer that could not be cured), unless findings pertaining to our population of interest were presented separately in the results section.
2. The studies did not report primary data (eg, systematic reviews, study protocols, conference abstracts, editorials, and commentaries).
3. The studies were not in the English language.

### Study Selection and Data Collection Process

In total, 2 authors (WG and MA) reviewed titles, abstracts, and full-text papers, assessing them for eligibility independently. Any disagreements were resolved through discussion.

Data from the included studies were extracted into a predesigned form by WG and verified by MA to capture study characteristics (design, sample size, cancer type, gender, age, and outcomes). Data were also extracted based upon the items included in the Template for Intervention Description and Replication checklist (why, what, who provided, how, where, when and how much, tailoring, modifications, and how well) [24].

### Quality Assessment

The included studies were assessed for methodological quality and risk of bias independently by 2 authors (WG and MA), with any disagreements resolved through discussion. The risk of bias for randomized controlled trials (RCTs) and nonrandomized studies was assessed using the Mixed Methods Appraisal Tool [25].

### Data Synthesis

A narrative synthesis [26] was conducted to outline the design, population, and context (mode of delivery, health care provider, and intervention intensity) of the individual studies. Studies were categorized by their approach to examining intervention effect, differentiating between those exploring pure intervention effect (eg, using blinded RCT designs) and those exploring effect in the context of routine health care [27]. For the primary outcome of engagement, a deductive and inductive approach was taken using the definitions of engagement behavior outlined by Perski et al [19] while also ensuring that other engagement-related data were captured. Engagement data were identified and split into categories based upon the type of engagement the studies measured: frequency (how often contact was made with the intervention over a specified period), the amount or breadth (the total length of each intervention contact), duration (the period over which participants were exposed to an intervention), and depth (variety of content used) [19]. Across these 4 measures, studies were grouped together based upon how they measured the outcome, which was then summarized.

Data from the included studies relating to frequency of use by patients, where reported, were transformed to a percentage of actual patient engagement compared with intended engagement with the intervention to provide a standardized statistical comparison. When overall engagement percentages were calculated, these were weighted by sample size.

To draw associations between the calculated percentage of actual patient engagement, the intensity of the intervention (for the patient and health professional), and mode of delivery, we had to simplify these characteristics. The intensity of the interventions for both the patient and the health professional was coded by a member of the research team (WG). WG reviewed the intervention description in each included study to determine the expected engagement with the intervention for patients and health professionals. This referred to any interaction (both scheduled and unscheduled) that was anticipated or planned with the intervention (eg, a patient having a telephone consultation with a health professional or submitting data via a web-based system). For articles where a second opinion was requested by WG, a second reviewer (MA) discussed the study with WG until a consensus was achieved on the expected engagement reported. The expected engagement was simplified into categories of high, medium, and low expected engagement to make comparisons across studies. For patients, low expected engagement referred to only having ≤3 contacts with the intervention, a medium level of engagement was 4 to 7 expected contacts, and a high level of engagement was ≥8 expected contacts or more than daily reporting of symptoms. A previous study of engagement with a web-based mindfulness intervention identified similar levels of high and low participant engagement (low: 0-4 and high: 5-7); however, a third category was added for this review to account for the studies with >7 contacts [28]. For health professionals, the categories mirrored those for patients if the health professional was required to make contact with the patient (eg, low was ≤3 contacts, medium was 4 to 7 contacts, and high was ≥8 contacts). If the health professional was required to only make contact with the patient when prompted to do so by a patient’s entry on a system or survey, it was coded as low contact on the part of the health professional. For each intervention, we also coded the mode of delivery (eg, telephone, smartphone, or web-based), including interventions where multiple modes were used. We were then able to look at associations between the mode of delivery, expected level of engagement (high, medium, or low for the patient and health professional), and the percentage of actual patient engagement with the intervention.

## Results

### Search Results

**Figure 1:**
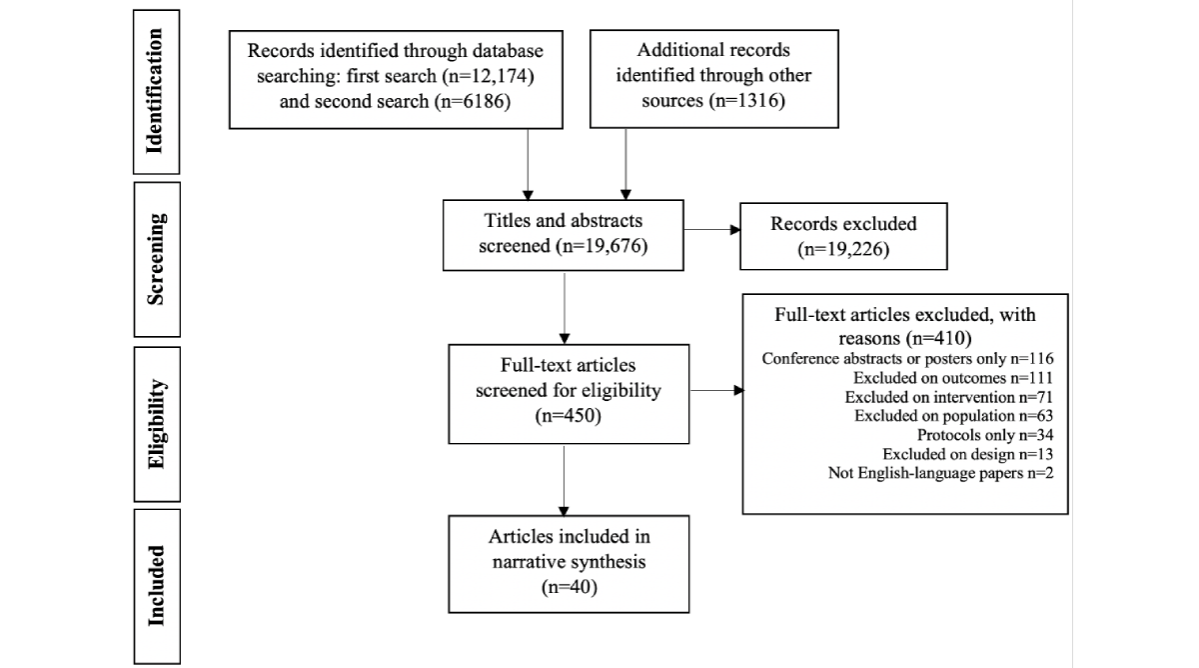
PRISMA (Preferred Reporting Items for Systematic Reviews and Meta-Analyses) flow diagram.

Of the 19,676 papers that were identified in the database search, 0.2% (40/19,676) of papers covering 39 different studies were eligible for inclusion in the systematic review [29-68]. Figure 1 outlines the PRISMA flow diagram for the included studies and the reasons for exclusion of studies.

### Study Characteristics

Table 1 includes a summary of the characteristics of the included studies. Table 2 outlines the characteristics of the included interventions and the engagement outcomes. The included studies had a sample size ranging from 6 [61] to 766 [31] and included multiple RCTs (16/39, 41%) [30,31,33-35,37-39,43-45,48,50,57,63,67,68], with most studies being conducted in the United States (22/39, 56%) [29-31,33,34,37-41,43,45,46,48,50,54,57,59,60,66-68]. Of the 39 studies included in the review, 13 (33%) explored intervention effects in the context of routine care implementation [29,32,36,41,42,49,51,52,58,60-62,64], with the remainder exploring intervention effects often using a blinded controlled trial design.

**Table 1:** Characteristics of the included studies (N=39).

Study	Country	Study design	Sample size	Type of cancer	Age (years)	Female participants, n (%)
Alter et al [29]	United States	Pilot	8	Colorectal	Range 59-79	5 (63)
Badr et al [30]	United States	RCT^a^	39	Lung	Mean 68 (SD 10)	29 (74)
Basch et al [31]	United States	RCT	IG^b^: 441; CG^c^: 325	Breast, genitourinary, gynecologic, or lung	IG: median 61; CG: median 62	IG: 257 (58); CG: 187 (58)
Bensink et al [32]	Australia	Feasibility	11	Advanced cancer, type NR^d^	Range 3-18	NR
Bouchard et al [33]	United States	RCT	192	Prostate	Mean 69 (SD 9)	0 (0)
Bruera et al [34]	United States	RCT	190	Advanced cancer, type NR	Median 58 (range 25-84)	128 (67)
Chambers et al [35]	Australia	RCT	189	Prostate	Mean 70 (SD 9)	0 (0)
Chavarri-Guerra et al [36]	Mexico	Observational study	45	Advanced cancer, type NR	Median 68 (range 33-90)	26 (58)
Cheung et al [37]	United States	RCT	39	Breast	NR	39 (100)
Cheville et al [38,39]	United States	RCT	516	Multiple myeloma, myelodysplastic syndrome, or lymphoma	Mean 66 (SD 11)	257 (50)
Chow et al [40]	United States	Feasibility	190	Advanced cancer, type NR	Median 68 (range 39-89)	94 (49)
Cluver et al [41]	United States	Feasibility	10	Advanced cancer, type NR	Mean 50 (range 26-61)	7 (70)
Dixon et al [42]	Canada	Feasibility	69	Advanced cancer, type NR	Mean 69	19 (28)
Donovan et al [43]	United States	RCT	65	Ovarian	Mean 57 (SD 9)	65 (100)
Eldeib et al [44]	Egypt	RCT	IG: 44; CG: 38	Colorectal or gastric adenocarcinoma	IG: mean 50 (SD 11); CG: mean 45 (SD 13)	IG: 28 (64); CG: 24 (63)
Flannery et al [45]	United States	RCT	IG: 30; CG: 15	Lung	IG: mean 66 (SD 8); CG: mean 61 (SD 9)	IG: 7 (41); CG: 5 (45)
Fleisher et al [46]	United States	Feasibility	22	Advanced cancer, type NR	Range 37-77	11 (50)
Fox et al [48]	United States	RCT	192	Prostate	IG: mean 71 (SD 8); CG: mean 71 (SD 9)	0 (0)
Fox et al [47]	Australia	Feasibility	15	Melanoma	26-49 years: n=4 (27%), 50-64 years: n=6 (40%), ≥65 years: n=5 (33%)	7 (47)
Friis et al [49]	Denmark	Feasibility	20	Lung	Median 70.5 (range 54-86)	7 (35)
Gustafson et al [50]	United States	RCT	IG: 144; CG: 141	Lung	IG: mean 62 (SD 11); CG: mean 61 (SD 10)	IG: 62 (50); CG: 59 (48)
Haddad et al [51]	Canada	Feasibility	IG: 102; CG: 118	Lung and others	IG: mean 62 (range 35-83); CG: mean 60 (range 31-87)	IG: 28 (50); CG: 25 (45)
Hennemann-Krauss et al [52]	Brazil	Observational study	12	Advanced cancer, type NR	Mean 68 (SD 9)	5 (42)
Keikes et al [53]	Netherlands	Feasibility	155	Colorectal	NR	NR
Liu et al [54]	United States	Pilot	16	Ovarian	Median 58 (range 36-80)	NR
Nemecek et al [55]	Austria	Feasibility	15	Non–small cell lung cancer, melanoma, and pancreatic	Mean 50	NR
Rasschaert [56]	Belgium	Feasibility	11	Colorectal, gastric or esophageal, pancreatic, and cholangiocarcinoma	Median 57 (range 44-74)	6 (55)
Rose et al [57]	United States	RCT	210	Advanced cancer, type NR	40-60 (n=109); 61-80 (n=101)	69 (33)
Sardell et al [58]	United Kingdom	Feasibility	45	Glioma	Median 50 (range 23-69)	15 (33)
Schmitz et al [59]	United States	Pilot	7	Breast	Mean 61	7 (100)
Sherry et al [60]	United States	Pilot	41	Lung	Mean 66 (SD 10)	29 (71)
Trojan et al [61]	Switzerland	Observational study	6	Prostate, lung, and urothelial	NR	0 (0)
Upton [62]	United Kingdom	Pilot	18	Melanoma	NR	NR
Voruganti et al [63]	Canada	RCT	IG: 24; CG: 24	Breast, colorectal, lung, prostate, ovarian, head and neck, and leukemia, myeloma, or lymphoma	IG: mean 60 (SD 13); CG: mean 60 (SD 14)	IG: 13 (62); CG: 16 (76)
Watanabe et al [64]	Canada	Pilot	44	Breast, lung, and leukemia, myeloma, or lymphoma	Median 60 (range 20-88)	18 (41)
Weaver et al [65]	United Kingdom	Pilot	26	Breast, colorectal	Mean 57	12 (46)
Wright et al [66]	United States	Pilot	10	Gynecologic	Mean 60 (SD 11)	10 (100)
Yanez et al [67]	United States	RCT	74	Prostate	Mean 69 (SD 9)	0 (0)
Yount et al [68]	United States	RCT	IG: 123; CG: 130	Lung	IG: mean 61 (SD 10); CG: mean 60 (SD 10)	IG: 66 (54); CG: 62 (48)

^a^RCT: randomized controlled trial.

^b^IG: intervention group.

^c^CG: control group.

^d^NR: not reported.

**Table 2:** Intervention details and engagement outcomes (N=39).

Study	Intervention intensity (duration of the intervention)	Intervention description (content, mode of delivery, health care provider)	Engagement outcomes (frequency, amount, duration, depth, and actual patient engagement)
Alter et al [29]	Four 30-minute telephone sessions (2 months)	Content: nurse gathered information on medical and psychological history and discussed effects of cancer on their lives and relationships. Concerns were identified and discussed, strengths in dealing with problems were also identified, and patients were encouraged to use strategies and resources that had been highlighted. Mode of delivery: telephone. Individual basis. Health care provider: nurse.	Frequency: all 4 patients completed all 4 telephone sessions. Actual patient engagement: 100%.
Badr et al [30]	Six 60-minute telephone sessions (6 weeks)	Content: a manual was used covering six areas: self-care, stress and coping, symptom management, effective communication, problem solving, and maintaining and enhancing relationships. Telephone sessions reviewed the content of the manual with patients and carers and set homework for following week. Mode of delivery: telephone. Patient–caregiver dyads. Health care provider: trained therapist in mental health counseling.	Frequency: 90% of patient–caregiver dyad phone calls were made on time. One member had scheduling conflicts, but all were made up with another call. Actual patient engagement: 100%.
Basch et al [31]	Participants remained in the study until treatment had concluded or they had died. All intervention participants reported symptoms on tablet or computer kiosks at clinic, but computer-literate participants also sent weekly emails to complete surveys at home (not set).	Content: participants who were computer-experienced completed symptom-tracking surveys in between clinic visits; if symptoms worsened, this would trigger an email alert to nurses, and participants were encouraged to call if concerned. Those who were computer-inexperienced completed surveys at the clinic before meeting with their clinician. Reports were provided to clinicians but no guidance on what action to take. Mode of delivery: computer or tablet. Individual basis. Health care provider: nurses and oncologists.	Frequency: 73% of intervention participants completed a symptom self-report at any clinic visit, but this did not lead to a difference in the number of nurse calls received compared with the control group (12.8 vs 12.9).
Bensink et al [32]	Individually tailored. No set engagement (not set).	Content: the families were provided with videoconference technology, which was used to provide patient assessment and monitoring, family education, communication, and counseling by nurses and other support by social workers or other medical staff. Mode of delivery: teleconference. Individual basis. Health care provider: nurses and social workers.	Frequency: 7 of 11 families received telephone calls, with a total of 25 made and an average of 2.3. Amount: calls lasted for a median length of 20 (IQR 15-33) minutes.
Bouchard et al [33]	Ten 90-minute group sessions (10 weeks)	Content: involved group teleconferences teaching stress and self-management skills for men with prostate cancer with disease-relevant examples. Mode of delivery: teleconference and telephone. Group delivery. Health care provider: therapist.	Frequency: an average of 7.5 (SD 3.1) sessions were attended for the intervention group. Actual patient engagement: 75%.
Bruera et al [34]	4-6 calls (2 weeks)	Content: the calls involved symptom assessment, a review of the types and dosages of medications and their effects, and psychosocial support and patient education. The patient could ask questions, and the nurse asked about their well-being. Mode of delivery: telephone. Individual basis. Health care provider: nurse.	Frequency: no significant difference in the number of phone calls received across any of the four groups: drug and intervention phone call (median 5, IQR 4-6), drug and control call (median 4, IQR 3-5), placebo and intervention phone call (median 5, IQR 4-6), and placebo and control call (median 4, IQR 4-5).
Chambers et al [35]	Eight 75-minute group sessions (8 weeks)	Content: an introductory call was used to prepare participants for the group call, and a workbook was used to also guide these group calls. The group calls encouraged peer interaction to support learning mindfulness skills and tackling challenges. Participants were encouraged to engage in 1 mindfulness meditation daily. Mode of delivery: teleconference. Group delivery. Health care provider: health professional.	Frequency: 28% (n=26) attended 0 sessions, 20% (n=19) attended 1 to 3 sessions, 22% (n=21) attended 4 to 7 sessions, and 30% (n=28) attended 8 sessions. Amount: the average length of a session was 85 (SD 12) minutes.
Chavarri-Guerra et al [36]	Individually tailored. No set engagement (not set).	Content: care needs assessments were administered remotely; the multidisciplinary team met to discuss intervention plans, which were then put to the patient. If acceptable, these were then conducted remotely. Mode of delivery: teleconference, telephone, and SMS text messaging. Individual basis. Health care provider: multidisciplinary team.	Frequency: 163 supportive care interventions were provided to 45 patients (median number of interventions per patient 3, range 1-13). Amount: 0-15 minutes: 38 (23.3%), 16-30 minutes: 58 (35.6%), 31-45 minutes: 37 (22.7%), >45 minutes: 29 (17.8%), (SMS text messaging): 1 (0.6%). Depth: psychological care: 54 (33.1%), pain and symptom control: 41 (25.1%), nutritional counseling: 20 (12.6%), physical therapy: 14 (8.5%), end-of-life care: 13 (7.9%), geriatric assessment: 8 (4.9%), advance directive completion: 8 (4.9%), psychiatric care: 5 (3%).
Cheung et al [37]	Five 1-hour sessions (5 weeks)	Content: each session taught participants 3 out of 8 skills (noticing positive events, capitalizing on or savoring positive events, gratitude, mindfulness, positive reappraisal, focusing on personal strengths, setting and working toward attainable goals, and small acts of kindness), and they were instructed to practice every day. Mode of delivery: web-based. Individual basis. Health care provider: unclear.	Frequency: all 12 participants completed 1 session, 11 participants completed 2 sessions, and 10 participants completed all 5 sessions.
Cheville et al [38,39]	8 telephone sessions with fitness care manager, 8 sessions with PT^a^ (more if PT thought needed), and pain management intervention arm received call from pain care manager, who then monitored patient-reported pain levels over the course of the study (4 weeks)	Content: intervention group 1: tele-delivery of rehabilitation services. Education on role of physical activity in symptom management, consequences of cancer and cancer treatment on loss of muscle bulk and power, and adverse symptoms during exercise. REST^b^ to improve functional status. FSP^c^ to increase activity levels and aerobic conditioning. Treatment of physical impairments (if any detected) through PT treatment plans. Intervention group 2: same as group 1 with additional pain management to monitor and adjust dosages and medication as needed. Mode of delivery: telephone and in person. Individual basis. Health care provider: primary care team, a PT acting as a fitness care manager, a physical medicine and rehabilitation physician, and a local physical therapist.	Frequency: no difference in remote monitoring contacts across the three groups: mean 10.3 (SD 4.4), mean 10.7 (SD 5.2), and mean 10.2 (SD 4.5). Contacts with the fitness care manager were similar across IG^d^ 1 and 2 (mean 7.6, SD 2.9, range 1-21 vs mean 7.2, SD 3.1, range 1-22). The proportion of surveys completed via the web as opposed to the IVR^e^ surveys was similar for each arm: CG^f^: 1648 (66%), IG 1: 1721 (74%), and IG 2: 1632 (69%). Amount: time spent with the fitness care manager was also similar across IG 1 and 2: mean 16.2 (SD 15.2, range 1-124) minutes for IG 1 and mean 16.6 (SD 15.4, range 1-87) minutes for IG 2. Actual patient engagement: IG 1: 95%; IG 2: 90%.
Chow et al [40]	5 telephone sessions (12 weeks)	Content: patients completed surveys on symptom distress, any questions were referred to palliative nurses, and clinic visits were only scheduled when necessary. Mode of delivery: telephone. Individual basis. Health care provider: health care professional trainee.	Frequency: of the 190 patients, 62% completed the week 1 and 2 phone call, 57% completed the week 4 phone call, 44% completed the week 8 phone call, and 40% completed the week 12 phone call. Actual patient engagement: 53%.
Cluver et al [41]	Six 60-minute sessions (not reported)	Content: sessions involved cognitive therapy. Mode of delivery: telephone and in person. Individual basis. Health care provider: therapist.	Frequency: of the 53 completed sessions, 21 were conducted via videophone, and 32 were conducted face-to-face. One session was missed.
Dixon et al [42]	2 telephone sessions (4 weeks)	Content: follow-up calls following radiation therapy were used to monitor patients’ symptoms. Mode of delivery: telephone. Individual basis. Health care provider: radiation therapist.	Frequency: 72% (38/53) of patients completed the telephone assessment at the 1- or 4-week intervals. Actual patient engagement: 72%.
Donovan et al [43]	Based upon participants’ engagement (3 weeks)	Content: patients had 3 target symptoms that they worked with the nurse to manage through the message board. The intervention encouraged the patient to understand their problem, discuss their concerns, and understand that they could make positive changes to manage their symptoms. Gaps in knowledge were addressed, and the benefits of new strategies were discussed as well as the setting of goals to achieve these. The patient was then followed up to see whether this worked or whether modifications needed to be made. Mode of delivery: web-based. Individual basis. Health care provider: nurse.	Frequency: the mean number of postings for the 33 women randomized into WRITE^g^ Symptoms was 15.87 (median 14, range 0-41). Amount: the mean length of participant posts was 260.50 (median 210, range 0-808) words. Duration: for those completing the intervention, it took the nurse–participant dyads an average of 79 (median 76, range 37-185) days to complete all elements of the intervention. Depth: 25 (75.8%) participants assigned to WRITE Symptoms completed all elements of the intervention.
Eldeib et al [44]	Weekly calls (dependent on length of treatment)	Content: phone calls were used to assess any adverse effects and recommend suitable strategies to remedy this. Adherence to medication was also reinforced. Mode of delivery: telephone. Individual basis. Health care provider: pharmacist.	Amount: total duration of calls was 1554 minutes; average of 35.3 minutes per patient (n=44).
Flannery et al [45]	8 telephone sessions (8 weeks)	Content: nurses phoned participants weekly and assessed their symptoms on 16 common symptoms experienced by those with lung cancer. Any reported symptom required asking questions about the somatic aspects of the symptom. Mode of delivery: telephone. Individual basis. Health care provider: nurse.	Frequency: of the 57% (17/30) of participants retained in the intervention arm, the mean number of intervention calls received was 5.50 (SD 2.48); 8 of 17 participants received all 8 interventions. Actual patient engagement: 68.8%.
Fleisher et al [46]	Dependent on participant engagement with web-based survey and skills module (not reported)	Content: a web-based survey on patient goals, values, and communication preferences, followed by a training module on communication skills. A report was generated for the physician to help guide their next session. Mode of delivery: web-based. Individual basis. Health care provider: oncologist.	Frequency: 18 began the communication aid, and 15 completed it. Amount: the average time for completing the entire program was 65 minutes—52 minutes spent on the survey and 13 spent on the module. Actual patient engagement: 83.3%.
Fox et al [48]	Ten 90-minute sessions (10 weeks)	Content: facilitator-led relaxation exercises (eg, deep breathing, progressive muscle relaxation, mindfulness meditation, and guided imagery). Psychoeducational sessions focused on stress management. Participants also given homework to practice skills learned in weekly sessions. Mode of delivery: web-based. Group delivery. Health care provider: therapist.	Frequency: week 1: 74% (n=70) attended IG meeting, and 75% (n=73) attended CG meeting. Week 10: 73% (n=69) attended IG meeting, and 82% (n=80) attended the CG meeting.
Fox [47]	1 telephone call (not set)	Content: the outreach call was tailored to the needs of the participant and considered their internal and external environments, including mental, physical, spiritual, psychological, cognitive, relational, social, and cultural aspects. Mode of delivery: telephone. Individual basis. Health care provider: social worker or counselor and nurse.	Amount: mean duration of calls was 56.5 (SD 15.72) minutes. Approximately 71% of calls lasted ≤1 hour.
Friis et al [49]	Once a week for 4-week web-based symptom reporting, telephone call if threshold exceeded (4 weeks)	Content: patients filled in health questionnaires in real time, which could be accessed by their health team. Those who needed clinical attention had alerts sent to the clinical team. Mode of delivery: web-based and telephone. Individual basis. Health care provider: nurse.	Frequency: 55% (37/67) of questionnaires answered exceeded the threshold and led to further action by a clinical nurse. Approximately 30% (20/67) of the questionnaires resulted in a phone call.
Gustafson et al [50]	Dependent on participant engagement (25 months long or 13 months after patient death for caregiver)	Content: access to Coping with Lung Cancer website, which provided information on lung cancer, care giving, and bereavement. It also acted as a communication channel between peers, experts, and clinicians. Feedback was also provided by algorithms based on collected data. Tools to help organize support were also provided. Clinicians received reports before next clinic appointments as well as email alerts when high symptom ratings were reported. Mode of delivery: web-based. Patient–caregiver dyad. Health care provider: oncologist and enrollment coordinator.	Frequency: CHESS^h^ was used at least once by 73.4% of caregivers and 50% of patients, and 51.6% of caregivers and 34.7% of patients used CHESS ≥5 times. Amount: the median number of minutes of CHESS use was 103 for caregivers and 146 for patients. Depth: the median number of pages viewed was 147 for caregivers and 243 for patients.
Haddad et al [51]	2 telephone sessions (4 weeks)	Content: participants were asked about their symptoms, side effects, and drug dosage. Mode of delivery: telephone. Individual basis. Health care provider: nurse and radiation therapist.	Frequency: successful contact at week 1 and 4 was achieved for 22 participants of group A, 14 participants only contacted at week 1, and 3 participants only contacted at week 4. A total of 17 participants were not contacted. Actual patient engagement: 54.5%.
Hennemann-Krause et al [52]	Web conferences weekly and face-to-face meetings monthly (continued until patient death)	Content: symptoms were assessed on a scale, and complaints from patients were listened to. In videoconferences, discrepancy between what the patients reported and what the physician could see onscreen were evaluated. Mode of delivery: teleconference, email, telephone, and in person. Individual basis. Health care provider: physicians, nurse, social worker, psychologist, and music therapist.	Frequency: in-person consultations: mean 7.42 (SD 6.29), web conferences: mean 6.42 (SD 7.64), and total contacts: mean 25.4 (SD 16.3). Duration: the mean monitoring time was 195 (SD 175.1) days.
Keikes et al [53]	2 face-to-face consultations and web-based access to decision support tool in between meetings (not reported)	Content: treatment options were discussed with oncologist, and the patient reviewed information available on the web and completed questions on treatment goals. Mode of delivery: web-based. Individual basis. Health care provider: oncologist and a helpdesk.	Frequency: 301 patients received a consultation sheet, of whom 155 patients participated in the web-based part of the decision tool (51%). Amount: the median overall time spent on web-based decision support was 38 (IQR 18-56) minutes. Time spent was highest on reading treatment background information (median 4, IQR 1-11 minutes) and answering questions about patients’ perspective (median 5, IQR 2-11 minutes). Actual patient engagement: 51%.
Liu et al [54]	Twice daily reporting of blood pressure and diarrhea data reported as needed. Algorithmic feedback and prompts to call HCP^i^ when appropriate (4 weeks).	Content: participants reported blood pressure and diarrhea entries, which triggered algorithmic feedback, and the clinical team reviewed this. Email alerts were sent to the clinical team for high results or when a blood pressure check was missed. Mode of delivery: mobile app. Individual basis. Health care provider: patients’ clinical team.	Frequency: patients using eCO^j^ recorded 98.2% of expected home blood pressure values. All 12 patients were prompted to call at least once, with most being prompted 7 to 20 times. One patient was prompted 54 times but was considered noncompliant. Actual patient engagement: 98.2%.
Nemecek et al [55]	Participant-dependent reporting and contact with physician (until participant death)	Content: VSee was used to connect patients and their physicians when the patient required medical advice. This was available around the clock. Patients could also input vital signs (temperature, blood pressure, pulse, and oxygen saturation) as well as treatment and other variables (pain, nutrition, and body weight). This could then be reviewed by the physician in charge. Mode of delivery: teleconference. Individual basis. Health care provider: physician.	Frequency: a total of 37 telemedical requests were submitted, of which 35 were successful, whereas 2 failed. A total of 638 data entries were performed. Entry count varied between 1 and 265 per patient.
Rasschaert [56]	Reported daily treatment intake, toxicity, and disease-related symptoms. Calls made when toxicity levels were high (no set duration; patients used for duration of oral anticancer agent).	Content: participants were asked to self-report disease-related symptoms and treatment toxicity via an app. This could be accessed by physicians and cancer care providers at clinic visits or when admitted to hospital. Alerts would be sent to caregivers or phone calls would be organized when high toxicities were reported, and the participants were also told to seek help. Mode of delivery: smartphone. Individual basis. Health care provider: data manager, physician, and other health care professionals.	Frequency: average daily compliance with registration of treatment intake was 91.2%. Duration: 5 patients used the coach >4 weeks (and only 1 used it for >12 weeks). Actual patient engagement: 91.2%.
Rose et al [57]	1 face-to-face meeting, 1 follow-up call. Patients could then contact the nurse 24 hours a day, 7 days a week at their convenience (2 months).	Content: the initial meeting occurred in the patient’s home and was to set goals for patient communications and shared decision-making. Coping and communication issues, strategies to address problems, and concerns and expectations were also discussed. Follow-up calls covered the multifaceted impact of cancer and treatment, preparing patients for future therapy or progression, identifying goals either personal or of treatment, identifying further needs of support, supporting positive emotions of oneself, encouraging independence and coping, optimizing social support, addressing practical problems, and referring patients for additional support. Mode of delivery: telephone, email, or in person. Individual basis. Health care provider: nurse.	Frequency: average number of monthly contacts was higher among middle-aged group (mean 2.6, SD 2.7) than among the older age group (mean 2.0, SD 1.2). Amount: average length of calls was 10-11 minutes. Duration: average of 62 days of access to intervention.
Sardell et al [58]	3 monthly telephone calls and 1 face-to-face clinic visit at the fourth month. Telephone calls continued if no recurrent or progressive disease (4 months but also participant-dependent).	Content: the telephone calls followed a semistructured script, which allowed patients to talk freely about their symptoms, how they were feeling, and any problems they had. More structured questions on their neurological status, medication, use of hospital services, return to work, and social activities followed. Mode of delivery: telephone. Individual basis. Health care provider: nurse.	Frequency: a total of 254 telephone calls were made, with a median of 4 calls per patient (range 1-14). Amount: median time on calls was 10 (range 2-10) minutes. Duration: median time was 6 (range 2-21) months.
Schmitz et al [59]	Daily app notifications to engage and 1 weekly phone call with navigator (12 weeks)	Content: participants received a daily prompt to interact with the app. The app asked a symptom question, which, when answered, prompted different facial expressions from the nurse avatar and different verbal responses. Navigator calls focused on reviewing symptoms and steps, which were compiled in a report and emailed to the clinical care team. Mode of delivery: mobile app and telephone. Individual basis. Health care provider: patient navigators.	Duration: average use of the tablet was 69.9 days for 7 participants.
Sherry et al [60]	Pamphlet and 1 telephone session (1-3 days)	Content: a personalized pamphlet was presented to the patient based upon problems they noted when completing a distress survey. This was followed up by a phone call a couple of days later to answer any questions and to check understanding. The coach offered referrals to social work, palliative and supportive care services, physical therapy, integrative medicine, financial services, and nutrition. Mode of delivery: telephone and in person. Individual basis. Health care provider: nurse.	Frequency: all patients reported that they had read the education pamphlet and received the coaching call.
Trojan et al [61]	Participant-dependent reporting of symptoms and side effects (3 months)	Content: patients reported the number, characteristics, and intensity of symptoms and therapy side effects. The symptom severity could trigger alerts to the on-call oncologist, which could result in a telephone consultation. Mode of delivery: mobile app and telephone. Individual basis. Health care provider: oncologist.	Frequency: 1279 symptom entries were recorded. Number of symptom data entries from the 6 patients ranged from 31 to 458 within the 3-month period. A total of 4 of the 6 patients also triggered 14 alerts, all of which correlated to cough, respiratory stress, fever, and fatigue and made patients aware of making contact with their treating center. A total of 6 alerts resulted in telephone consultations with the treating center or oncologist on call.
Upton [62]	1 telephone assessment (1 day)	Content: before ipilimumab infusion, the patient’s blood was tested, and immune-related adverse events were assessed by the nurse. After the infusion, patients were contacted weekly to monitor for immune-related adverse events and for the nurse to provide advice. Patients were also asked to call a 24-hour triage service if experiencing any problems. Mode of delivery: telephone and in person. Individual basis. Health care provider: nurse.	Frequency: over a 1-year period, a total of 56 telephone assessments were undertaken.
Voruganti et al [63]	Dependent on participant engagement with web-based messaging and communication with HCPs (not reported)	Content: the web-based communication tool (Loop) facilitated conversations between patients, caregivers, and health care providers. There was no set communication the tool should be used for, only that it should not be used for urgent communication. Mode of delivery: web-based. Individual basis. Health care provider: oncologist, palliative care physician, and other health care professionals.	Frequency: over the study period, most (17/20, 85%) Loops (web-based tool to facilitate communication) had message exchanges, with 65% (13/20) having >6 messages exchanged. During the study, there were 358 log-ins by all participants: 43 on the mobile version and 315 on the desktop version.
Watanabe et al [64]	One 90-minute videoconference with a 30-minute follow-up if necessary (1 day)	Content: patients arranged to attend a local clinic, where a videoconference could be set up with the cancer institute. Blood tests, radiological investigations, and patients’ symptoms and needs were assessed before this, and the results were shared with the team. A total of 3 team members, including the physician, could be on the videoconference, with every member given 15 minutes to interview the patient. After the assessments, the team formed a management plan in discussion with the patient and family, which was sent to the patient’s GP^k^. Mode of delivery: teleconference. Individual basis. Health care provider: nurses, dieticians, psychologists, respiratory therapists, social workers, occupational therapists, physical therapists, speech language pathologists, radiation oncologists, and pharmacists.	Frequency: a total of 72 clinic visits took place, consisting of 44 initial consultations and 28 follow-up visits. Depth: variety of members of MDT^l^ seen at consultations: dieticians (56.8%), psychologists (27.3%), respiratory therapists (15.9%), social workers (13.6%), occupational therapists (9.1%), physical therapists (9.1%), and speech language pathologists (4.5%). Actual patient engagement: 100%.
Weaver et al [65]	Phone app used twice daily to report symptoms; alerts to nurse generated if toxicity was high or the patient had not self-reported for a while (while on treatment)	Content: patients asked to fill out a short diary containing entries for temperature, diarrhea and assessments for vomiting, nausea, mucositis, hand–foot syndrome, and—for patients receiving oxaliplatin—peripheral neuropathy. Alerts were triggered based upon toxic side effects or a lack of reporting, with a nurse available to provide clinical advice. Mode of delivery: mobile app. Individual basis. Health care provider: nurse.	Frequency: the patients completed the diary on 92.6% of occasions (range 73.7%-100%). On 396 occasions, self-care advice messages were sent to the patients. Actual patient engagement: 92.6%.
Wright et al [66]	Daily app notifications for 30 days. If high-risk symptoms were reported, the patient was told to contact the clinician (30 days).	Content: participants completed daily surveys on quality of life, physical function, and symptoms, of which they ranked the severity. High-risk symptoms initiated a prompt to contact the participant’s clinician with an in-built call button. Mode of delivery: mobile app and telephone. Individual basis. Health care provider: oncologists and researchers.	Frequency: study participants were 70% adherent to smartphone surveys. A total of 7 participants answered daily surveys ≥4 times a week. Actual patient engagement: 70%.
Yanez et al [67]	Ten 90-minute group sessions (10 weeks)	Content: participants were taught a stress reduction or relaxation technique while also developing stress awareness, learning stress reduction skills, changing negative stressor appraisals, developing coping skills, building interpersonal skills, and building or enhancing social networks. They were also encouraged to access the website, which contained material related to each group session and videos to review in between sessions. Mode of delivery: teleconference. Group delivery. Health care provider: therapists.	Frequency: HP^m^ participants completed significantly more sessions (mean 8.22, SD 2.75 compared with mean 6.59, SD 3.72) than CBSM^n^ participants. HP participants also completed significantly more weekly assessments (mean 7.05, SD 3.14) vs mean 4.84, SD 3.35) compared with the CBSM condition. Actual patient engagement: 65.9%.
Yount et al [68]	Weekly calls to report symptoms, alerts triggered calls from a nurse (12 weeks)	Content: participants completed a symptom survey over the phone using the telephone keypad. Clinically significant symptoms were automatically reported to the clinical team for assessment and management with a nurse phone call. Data were also provided to physicians every 3 weeks before visits to facilitate discussion. Mode of delivery: telephone. Individual basis. Health care provider: physicians.	Frequency: compliance with completion of weekly symptom monitoring phone calls was 82.1%. Actual patient engagement: 80.8%.

^a^PT: physical therapist.

^b^REST: Rapid Easy Strength Training.

^c^FSP: First Step Program.

^d^IG: intervention group.

^e^IVR: interactive voice response.

^f^CG: control group.

^g^WRITE: Written Representational Intervention To Ease Symptoms.

^h^CHESS: Comprehensive Health Enhancement Support System.

^i^HCP: health care professional.

^j^eCO: eCediranib/Olaparib.

^k^GP: general practitioner.

^l^MDT: multidisciplinary team.

^m^HP: health promotion.

^n^CBSM: cognitive behavioral stress management.

### Engagement

The engagement outcomes for all studies are outlined in Table 2.

#### Frequency

Across most studies (36/39, 92%), the frequency of times contact was made with the intervention was reported [29-43,45-47,49-58,60-68]. There was substantial heterogeneity in the measurement of frequency across studies. Of the 39 studies, 13 (33%) reported the percentage of contacts either with the whole intervention or with each individual intended session [30,31,35,40,48-50,54,56,63,65,66,68]. The number of contacts with the intervention overall or each individual session was reported by 69% (27/39) of the studies [29,33,34,36-39,41-43,45,46,49,51-55,57,58,60-67].

Across 44% (17/39) of studies, it was possible to create a standardized percentage of actual patient engagement compared with intended engagement [29,30,32,33,38-40,42,45,46, 51,53,54,56,63,66-68]. This ranged from 51% [53] to 100% [29,30,64], with an average across all 17 studies of 75.4% (SD 15.8%). In the remaining 49% (19/39) of studies, it was not possible to create this standardized statistic because of a lack of reported data, and the design of the intervention meant there was no intended engagement and it was instead tailored to the patients’ needs.

#### Amount

A total of 31% (12/39) of studies measured the amount of contact with each intervention or with the intervention overall [32,35,36,38,39,43,44,46,47,50,53,57,58]. Of the 39 studies, 3 (8%) measured the average amount of time of each intervention contact (10.5 to 85 minutes) [35,47,57], 2 (5%) reported the average amount of time across all intervention contacts (16 to 65 minutes) [38,39,46] and 1 (3%) reported the total amount of call durations, which could be averaged across all intervention participants to 35.3 minutes [44]. In total, 5% (2/39) of studies reported the median amount of time for each intervention contact (10 to 20 minutes) [32,58], and 5% (2/39) of studies reported the median amount of time across the whole intervention (38 to 146 minutes) [50,53]. A total of 3% (1/39) of studies reported the number of intervention contacts that fell into a range of minutes (eg, 16-30 minutes: 58 contacts) [36]. A total of 3% (1/39) of studies did not report time but, as it was a web-based intervention with communication with the health professional through posts on a message board, instead reported the average length of each post at 260.5 words [43].

#### Duration

A total of 15% (6/39) of studies that had open-ended interventions reported the length of time that each participant was exposed to the intervention [43,52,56-59]. A total of 10% (4/39) of studies reported the average time of exposure to the intervention, ranging from 62 to 195 days [43,52,57,59]. A total of 3% (1/39) of studies reported a median amount of exposure to the intervention of 6 months [58], and the final study (1/39, 3%) reported the number of participants exposed for >4 weeks (n=5) and >12 weeks (n=1) [56].

#### Depth

A total of 10% (4/39) of studies reported on the variety of components of the intervention that the participants accessed [36,43,50,64]. Each study measured depth in different ways. A total of 3% (1/39) of studies reported the percentage of time that each health professional was on the teleconference calls [64], and another study (1/39, 3%) simply reported that 75% of patients had completed all elements [43]. The number of different interventions that all participants received was reported by 3% (1/39) of studies [36], and the final study (1/39, 3%) reported that patients had viewed a median of 243 webpages [50].

### Association With Intervention Level of Intensity

Expected levels of engagement for both patients and health professionals were reported across low (≤3 contacts), medium (4-7 contacts), and high (≥8 contacts) categories. A total of 13% (5/39) of studies could not be categorized as there was no expected engagement with the intervention, and the extent of engagement was determined at the patient’s discretion [32,36,43,55,63]. Table 3 shows the number of studies with the expected interaction of both the patient and health professional with the intervention. Most studies expected a similar level of interaction from both the patient and health professional in an intervention, but no studies expected more interaction from the health professional than from the patient.

**Table 3:** Number of studies with the expected engagement of the patient and health professional (n=34).

Expected patient interaction with the intervention	Expected health professional interaction with the intervention		
	Low, n (%)	Medium, n (%)	High, n (%)
Low	10 (26)	—^a^	—
Medium	2 (5)	8 (21)	—
High	7 (18)	—	7 (18)

^a^No data available for category.

**Figure 2:**
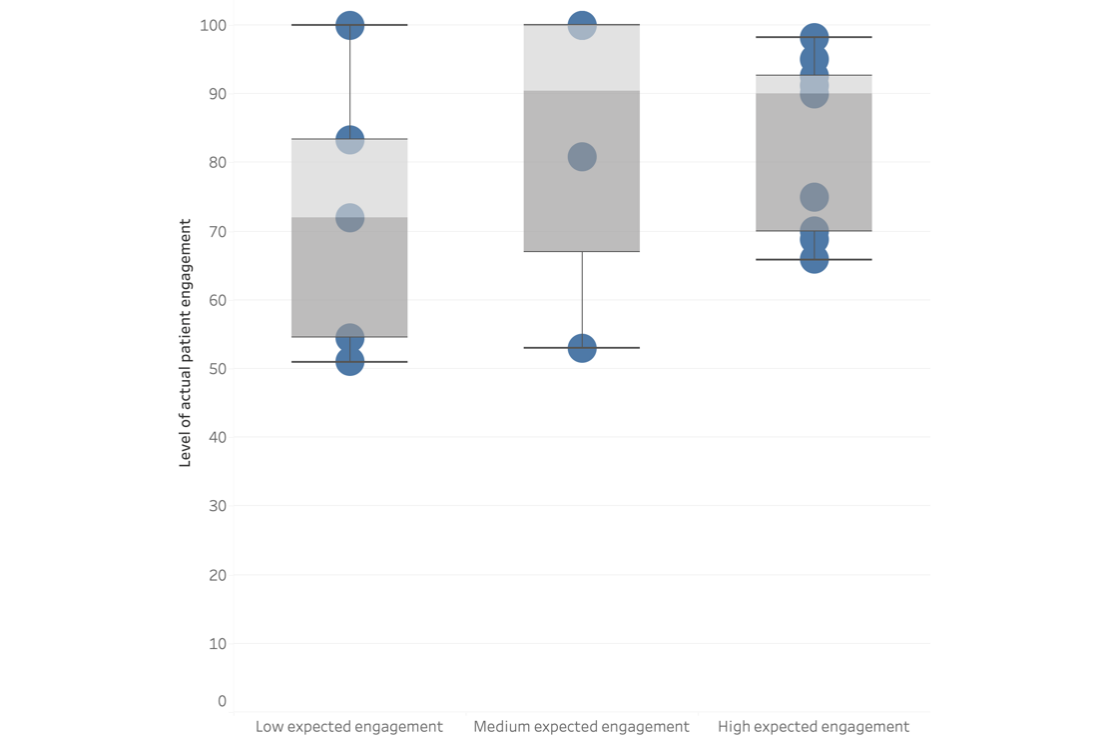
Box plot to present the association between expected levels of engagement by the patient and the percentage of actual engagement by the patient.

**Figure 3:**
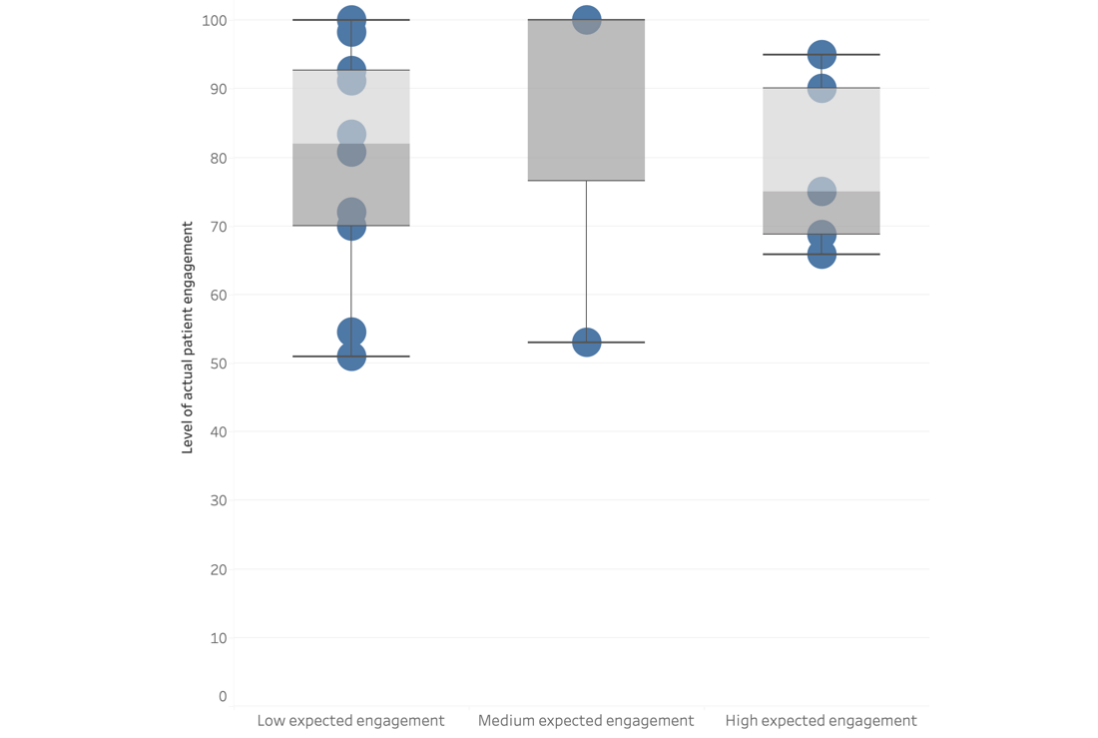
Box plot to present the association between expected levels of engagement by the health professional and the percentage of actual engagement by the patient.

Figures 2 and 3 are graphical representations of the association between expected levels of engagement for the patient (Figure 2) and the health professional (Figure 3) and the percentage of actual engagement with the intervention by the patient. Figure 2 shows that the studies that had low expected engagement for the patients had a combined actual patient engagement of 64% (SD 14.8%); for medium expected engagement, this was 66.9% (SD 16.4%); and, for high expected engagement, this was 87% (SD 8.2%). Figure 3 shows that the category with the highest level of combined actual patient engagement was the studies that expected the health professionals to have a high level of engagement with the intervention (86.6%, SD 8.3%). The studies in the categories of low and medium expected engagement from health professionals had lower levels of combined actual patient engagement (71%, SD 15.2% and 62.3%, SD 15%, respectively).

### Association With Intervention Mode of Delivery and Health Care Providers

**Figure 4:**
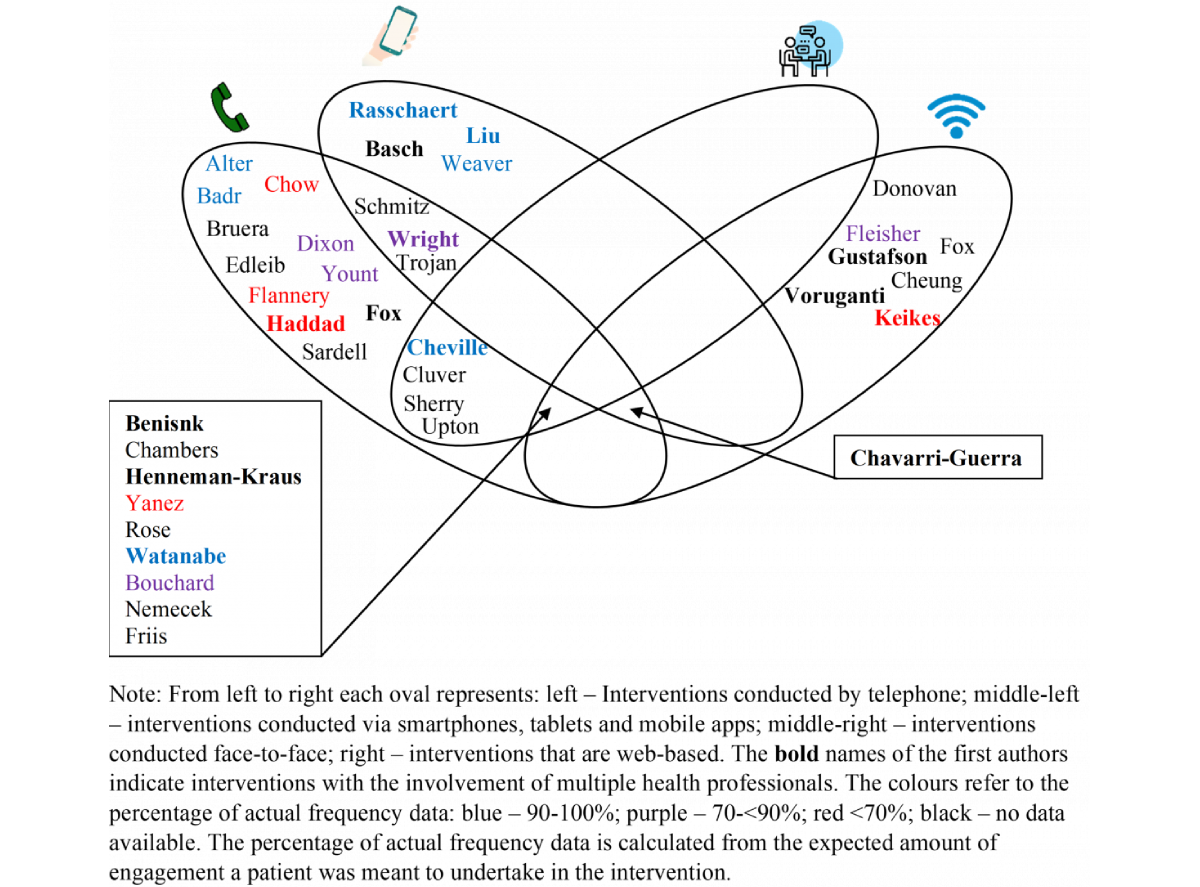
Modes of delivery of each intervention and, where reported, the percentage of actual frequency of engagement [29-68].

Figure 4 [29-68] shows the modes of delivery of each intervention and where interventions use multiple modes, with the names in bold involving multiple health professionals. The figure also shows, where available, the percentage of actual patient engagement by way of color, with blue showing 90% to 100%, purple showing 70% to 89%, and red showing <70%. Of the 39 studies, 17 (44%) used multiple modes of delivery, whereas the remaining 22 (56%) used 1 mode. The telephone was the most popular mode of delivery (28/39, 72%) followed by web-based delivery of the intervention (17/39, 44%). The use of only a tablet or smartphone app for the intervention appeared to be associated with the most actual patient engagement with an intervention, with 8% (3/39) of studies showing between 90% and 100% engagement [54,56,65]. The use of a telephone was more mixed, with actual patient engagement ranging from 54.5% [51] to 100% [29,30]. Figure 4 also shows broadly how many health care providers were involved in delivering the interventions, with those involving multiple health care providers shown in bold. Those interventions that involved multiple health care providers reported higher patient engagement than those with only 1 health care provider (79.3%, SD 18.5% vs 70.5%, SD 11.5%).

### Study Quality

The included studies could be grouped into two broad categories to be assessed using the Mixed Methods Appraisal Tool: quantitative RCTs and quantitative nonrandomized trials. The RCTs were of a broadly high quality; however, a number of studies did not provide enough information to assess whether the randomization procedure was conducted adequately or whether the groups at baseline were comparable. There were also 15% (6/39) of studies that did not have complete outcome data at follow-up. Among the nonrandomized trials, study quality was again high, apart from the included studies that did not control for confounders in their analysis. This is likely because most of these studies were feasibility or pilot studies and were not powered to detect significance, which would have been inappropriate. A breakdown of how each study was rated can be found in Multimedia Appendix 2 [29-68].

## Discussion

### Principal Findings

This systematic review is the first to synthesize engagement data from telehealth interventions for people with advanced cancer. This review found that people with advanced cancer were able to successfully engage in telehealth interventions with variable types of telehealth modalities, including telephone, mobile phone–based apps, and web-based interventions, albeit largely in the context of research studies. This review found that the frequency of engagement with the intervention was the most commonly reported measure of engagement, although there was heterogeneity in the method of reporting across the studies. Where standardized comparison was possible across the studies, actual engagement as a proportion of intended engagement was at an average of 75.4% (SD 15.8%). The level of engagement was found to vary based on the expected interaction of both the patient and health care professional and the mode of delivery. Actual patient engagement was higher in studies that expected higher levels of engagement from both the patient and health care professional but was noticeably lower in studies that expected only a low or medium level of engagement. Furthermore, the use of only a tablet or smartphone app for an intervention appeared to be associated with the highest levels of actual patient engagement with an intervention. This could in part be explained by the immediacy of access and reduced steps for accessing an intervention through a mobile phone app when compared with an intervention hosted on a website.

This review is in line with previous reviews that looked at engagement with interventions involving digital technology among people with chronic diseases, which found broadly that there are high levels of engagement with interventions [20]. However, this review provides an overview and critique of existing reporting of engagement for telehealth interventions in patients with advanced cancer and found wide disparities in metrics for engagement used and reported across the included studies. The frequency of interaction with an intervention was reported widely, but other measures of engagement, such as the amount of time spent engaging with the intervention, were not reported as well. Furthermore, the duration and depth of engagement with the intervention were reported by only one-quarter of all included studies (9/39, 23%). This may be due to the design of interventions with a set duration or only 1 component that patients could engage with, but this was not clear across studies. In addition, few studies reported the expected levels of engagement for an intervention, limiting the interpretability of any subsequent reporting of actual patient engagement. Refining and using measures to better understand factors driving digital engagement, including for telehealth, could inform the development of approaches from design through monitoring as part of routine care. For example, the application of engagement measures could serve as a progression criterion in feasibility studies of emerging telehealth approaches. Future research may need to define and develop meaningful and context- and condition-appropriate measures of digital engagement for palliative care to facilitate measurement of digital engagement. Although this review focused on the quantitative measures of behavioral engagement, the future development of a measure should attempt to incorporate components that provide a broader understanding of subjective experiences and aspects of engagement, potentially through qualitative approaches. There is also scope to develop and refine the dimensions comprising the digital engagement framework used to guide the synthesis of data in this study. For example, there is scope to incorporate a temporal element to consider the intensity of the intervention (eg, whether the intervention is spread over a week or months) alongside refining the underpinning definitions of terminology used for each dimension as the framework continues to evolve.

Through this review, we can conclude that there is no standardized method to report engagement in telehealth interventions for people with advanced cancer. The frequency of interactions with the intervention was presented most commonly, although the way in which this was done varied greatly across the studies, and there is a limited ability to understand what this means in the context of the intervention and the proposed and expected engagement needed for clinical utility. For example, people with advanced cancer have fluctuating needs, and a higher level of engagement with an intervention may not relate to the success of the intervention itself but be reflective of worsening outcomes for the patient [69]. In addition, patients may have their symptom management needs met early on in the intervention and may not need further follow-up, which may not be indicative of poor engagement with the intervention per se. With regard to mobile health interventions, the Mobile Health Evaluation, Reporting and Assessment checklist has been developed to help standardize the methodology for reporting the content and context of an intervention to support reproducibility and comparison of interventions [70]. Future iterations of the tool could include, for example, reporting of the expected and actual patient engagement levels of intended users of telehealth interventions alongside frequency of use—the most widely reported measure in this review. These data could complement and contribute to emerging evidence regarding the feasibility and acceptability of telehealth approaches as part of care for people with advanced cancer.

Recent evidence suggests that digital health interventions could provide a degree of efficacy related to QOL and psychosocial well-being [16]. For this review, most included interventions focused on symptom management, with high levels of engagement that suggest potential for its use to support remote monitoring. This approach could facilitate reductions in the required number of in-person visits while enabling continued access to data to inform patient care. However, in order to ensure such an approach is sustainable, there is a need to consider the burden of data entry on patients and the need for review—and potentially response—by health professionals. For patients, emerging approaches provide options for enhancing the richness of data received through remote monitoring without increasing the data burden for patients. For example, wearable technologies can passively collect sensor data on heart rate and activity to inform automatic monitoring and feedback processes [71], augmenting existing approaches without increasing the need for manual data entry. For health professionals, this review found that studies with high levels of intended engagement for both the patient and health care professional were associated with higher levels of actual engagement on the part of the patient. High intended engagement from health care professionals may not be a sustainable approach for digital technology, particularly when considered alongside the additional *invisible* work that such digital health can create for health professionals (eg, data must be interpreted, made sense of, located within existing knowledge and data sets, and negotiated) [72]. This is important to consider in light of projections of an increasing burden of serious health-related suffering and subsequent demands on palliative care services across geographical regions where demand is increasingly outstripping supply [73,74]. Therefore, for telehealth approaches to be sustainable as part of care for people with advanced cancer, they should seek to balance demands on both the patient and the care team, seeking to achieve maximal information with minimal data burden.

### Limitations

There were a number of limitations associated with this review. First, the focus of engagement in this review was on the behavioral aspects that were outlined by Perski et al [19] but not on the subjective measures of engagement, such as interest, attention, and enjoyment. Integrating these subjective measures into a future mixed methods review could allow us to evaluate the experience of interventions. In addition, because of the heterogeneity of the studies and reported approaches to measuring engagement, such as frequency, it is difficult to determine exactly which components of interventions contribute to higher engagement levels. We were only able to draw associations, and future research is needed to better explore causal factors. Furthermore, although this review looked at the extent of engagement, how it was measured across studies, and the association with the study characteristics, we did not assess whether engagement led to an improvement in patient-reported outcomes or experience. A future review should consider how engagement interacts with patient-reported outcomes. In addition, when determining the categories for low, medium, and high expected engagement, we did not take into account the time frame of the intervention; therefore, 2 studies could be grouped together with different levels of intervention intensity. Furthermore, most of the studies included in this review explored the intervention effect through mostly controlled studies, which could bias the recruitment toward those individuals who were motivated and more likely to be technologically literate. The levels of engagement identified in this review may not then translate into routine clinical care if these studies and their intervention effect have to date been confined to exploration in the context of RCTs and similar study approaches. This review also limited the included studies to those written in English; therefore, this review may not contain the entirety of related studies.

### Conclusions

This review identified that, where reported, there is a high level of engagement with telehealth interventions among people with advanced cancer. We identified that actual patient engagement is associated with both the expected level of engagement of the patient and the health professional as well as the mode of delivery of the intervention. We highlighted the heterogeneity in the reporting of engagement results across the research and the need to improve such reporting guidelines. As treatment delivery becomes increasingly more dependent on remote or telehealth modalities, the inclusion of a measure of engagement in future telehealth evaluations is essential to enable the comparisons of interaction and use across intervention approaches and to provide further granularity in factors that determine optimal implementation of telehealth approaches. There is a need for consistent measurement and reporting of domains relating to digital engagement (eg, breadth, duration, and frequency) with the scope to amend or develop measures. This will increase the ease of reporting of engagement in future studies, inform which telehealth intervention components are linked to variations in engagement, facilitate evidence syntheses, and support the development of condition-specific benchmarks of digital engagement for people with advanced cancer.

## Appendix

Multimedia Appendix 1 Search strategy for Ovid MEDLINE.

Multimedia Appendix 2 Quality appraisal tables of quantitative randomized controlled trials and nonrandomized trials.

## Abbreviations

PRISMA: Preferred Reporting Items for Systematic Reviews and Meta-Analyses
PROSPERO: International Prospective Register of Systematic Reviews
QOL: quality of life
RCT: randomized controlled trial
