# Supplementary material for: The Extent of Engagement With Telehealth Approaches by Patients With Advanced Cancer: Systematic Review
Source: JMIR Cancer. 2022 Feb 17;8(1):e33355. doi: 10.2196/33355 (PMC8895292; doi:10.2196/33355)
Supplement: Multimedia Appendix 1 [file cancer_v8i1e33355_app1.docx]

**Supplementary Material 1 – Search strategy for Ovid MEDLINE**

1 exp neoplasms/ (3074768)

2 (cancer* or neoplasm* or malignan* or oncology or tumo?r* or sarcoma* or carcinoma* or leuk?emia* or lymphoma* or mesothelioma* or pseudomyxoma* or glioblastoma*).tw. (2820458)

3 1 or 2 (3621613)

4 palliative care/ or exp terminal care/ or hospices/ (89762)

5 Terminally Ill/ (6178)

6 exp Advance Care Planning/ (8343)

7 ("end of life" or end-stage or "non-curable" or palliative or hospice* or incurable or inoperable or untreatable or metasta* or "life limiting" or supportive).tw. (545852)

8 or/4-7 [EoL terms] (595110)

9 3 and 8 (432547)

10 ((chronic or advanced or secondary or stage-IV or stage-4 or terminal* or recurren* or relaps* or unresect*) adj4 (cancer* or neoplasm* or malignan* or oncology or tumo?r* or sarcoma* or carcinoma* or leuk?emia* or lymphoma* or mesothelioma* or pseudomyxoma*)).tw. (258424)

11 9 or 10 [advanced cancer] (621904)

12 exp Telecommunications/ (81399)

13 (telemedicine or tele-medicine or telehealth or tele-health or teleoncology or tele-oncology or telemonitor* or tele-monitor* or telecare or tele-care).tw,kw. (11507)

14 (ehealth* or e-health* or "electronic health*" or mhealth* or m-health* or "mobile health*").tw,kw. (14906)

15 (remote* adj3 (monitor* or consultation* or communication)).tw,kw. (2397)

16 ("smart phone*" or smartphone* or iphone* or "cell* phone*" or cellphone* or "mobile phone*" or ipad* or app? or mobile application?).tw,kw. (32584)

17 (PDA or handheld or hand-held or "instant messag*" or "text-messag*" or "electronic messag*" or "short messag* service" or phone messag* or texting or SMS or "web 2.0" or "health 2.0" or "medicine 2.0" or email*).tw,kw. (27045)

18 exp microcomputers/ (19431)

19 mobile applications/ or user-computer interface/ or web browser/ (38556)

20 (Skype or facetime or google or google+ or "google +" or "Google plus" or facebook or face-book or blog* or vlog* or videoblog* or YouTube or you tube).tw,kw. (12937)

21 VIDEO GAMES/ or exp VIDEO-AUDIO MEDIA/ or exp VIDEO RECORDING/ (59272)

22 (webcast* or web-cast* or podcast* or pod-cast* or videocast or video-cast or instagram or whatsapp or snapchat or WeChat or twitter or tweet*).tw,kw. (2481)

23 exp Accelerometry/ (6564)

24 (fitbit or "Apple watch*").tw,kw. (157)

25 (wear* adj5 (device* or monitor* or sensor* or technolog*)).tw,kw. (4113)

26 ((smart or digital or symptom*) adj3 track*).tw,kw. (350)

27 Virtual Reality Exposure Therapy/ or Virtual Reality/ (824)

28 (cyber or avatar* or audiovisual or audio-visual or second-life or virtual reality or multimedia).tw,kw. (15085)

29 (social media or social network* or social software or chat room* or chatroom* or crowdsourc* or crowd sourc* or cyworld).tw,kw. (16136)

30 (e-PRO* or ePRO* or E-PROM* or EPROM*).tw,kw. (5791)

31 ((electronic* or online or web* or internet or digital*) adj8 (self-report* or patient-report*)).tw,kw. (1660)

32 exp electronic health records/ or health smart cards/ (15461)

33 exp internet/ or Crowdsourcing/ (70274)

34 (bebo or dailystrength or doximity or dropbox or flickr or friendster or gaming or hi5 or hyves or igoogle or instagram or linkedin or myspace or myfamilyhealth or netvibes or orkut or pageflakes or patientslikeme).tw,kw. (2197)

35 (picasa or pinterest or plaxo or reddit or renren or researchgate or rss or sciencestage or screencast or slideshare
or studiviz or tumblr or vimeo or vodcast or widget or wiki or wordpress).tw,kw. (1913)

36 ((online or web or website or web-site or web-based or internet or digital or electronic or video or telephone) adj4 (technolog* or intervention* or peer* or support or group* or program* or monitor* or educat* or communicat* or resource* or tool*)).tw,kw. (51795)

37 or/12-36 [online interventions] (373992)

38 Electronic Palliative Care Coordination System*.tw,kw. (2)

39 Electronic Palliative Care Co-ordination System*.tw,kw. (0)

40 Comprehensive Health Enhancement Support System.tw. (45)

41 electronic Symptom Management using the Advanced Symptom Management System.tw,kw. (2)

42 (EMOSAIC or E-MOSAIC or E-rapid or ERAPID or EPACCS or eSMART or e-SMART or ASyMS).tw,kw. (39)

43 (CHESS and cancer).tw. (40)

44 or/38-43 [named systems] (102)

45 11 and (37 or 44) (3946)

46 exp Cells, Cultured/ (1520522)

47 exp animals/ not (exp animals/ and human/) (4488729)

48 Comment/ or Editorial/ or news/ or newspaper article/ (1142905)

49 (Comment* or editorial? or note?).ti. (118928)

50 or/46-49 (6659382)

51 45 not 50 (3369)

52 Economics/ (26947)

53 exp Economics, Dental/ (4041)

54 exp Economics, Nursing/ (3981)

55 exp Economics, Medical/ (14037)

56 exp Economics, pharmaceutical/ (2794)

57 exp Economics, Hospital/ (23028)

58 exp "Costs and Cost Analysis"/ (217678)

59 exp "Fees and Charges"/ (29374)

60 exp budgets/ (13345)

61 exp "Value of Life"/ec [Economics] (244)

62 budget*.tw. (21418)

63 cost*.ti. (94392)

64 (cost* adj2 (effective* or utilit* or benefit* or minimi* or evaluat* or analy* or study or studies or consequenc* or compar* or efficienc* or variable or unit or estimate* or variable* or unit)).ab. (129731)

65 (economic* or pharmacoeconomic* or pharmaco-economic*).tw. (191296)

66 (price or prices or pricing).tw. (28553)

67 (financ* adj2 (cost* or data or "health care")).tw. (6736)

68 (fee or fees).tw. (14265)

69 (value adj1 (money or monetary)).tw. (464)

70 quality-adjusted life years/ (10354)

71 (eq-5d* or eq5d* or euroquol* or euroqol* or euroqual* or euro-quol* or euro-qol* or euro-qual*).tw. (6985)

72 exp models, economic/ (13478)

73 markov chains/ (12919)

74 quality adjusted life.tw. (9011)

75 (qaly or qalys or qald or qale or qtime).tw. (7309)

76 disability adjusted life.tw. (2112)

77 (daly or dalys).tw. (1923)

78 "Global Burden of Disease"/ [new 2017] (158)

79 health* year* equivalent*.tw. (38)

80 (hye or hyes).tw. (57)

81 (hui1 or hui2 or hui3).tw. (316)

82 disutil*.tw. (321)

83 standard gamble*.tw. (738)

84 (time trade off or time tradeoff).tw. (1145)

85 (hqol or h qol or hrqol or hr qol).tw. (11761)

86 (pqol or qls).tw. (292)

87 (sf6d or sf 6d or short form 6d or shortform 6d or sf sixd or sf six d).tw. (631)

88 Incremental cost effectiveness analysis.tw. (164)

89 Incremental cost effectiveness ratio.tw. (3561)

90 Net present value*.tw. (443)

91 Incremental net benefit*.tw. (99)

92 ec.fs. (395617)

93 net monetary benefit*.tw. (190)

94 or/52-93 (758365)

95 exp animals/ not (exp animals/ and exp humans/) (4488729)

96 exp Veterinary Medicine/ (24277)

97 exp Animal Experimentation/ (8786)

98 ((energy or oxygen* or metaboli*) adj3 (expenditure* or cost*)).tw. (26734)

99 or/95-98 (4526866)

100 94 not 99 (712905)

101 51 and 100 [economic evaluations] (197)
