# Supplementary material for: The Extent of Engagement With Telehealth Approaches by Patients With Advanced Cancer: Systematic Review
Source: JMIR Cancer. 2022 Feb 17;8(1):e33355. doi: 10.2196/33355 (PMC8895292; doi:10.2196/33355)
Supplement: Multimedia Appendix 2 [file cancer_v8i1e33355_app2.docx]

**Supplementary Material 2 – Quality appraisal tables of quantitative randomised controlled trials and non-randomised trials.**

| Study | **MMAT – Quantitative randomised controlled trials** | | | | |
| --- | --- | --- | --- | --- | --- |
|  | Is randomisation appropriately performed? | Are the groups comparable at baseline? | Are there complete outcome data? | Are outcome assessors blinded to the intervention provided? | Did the participants adhere to the assigned intervention? |
| Badr, 2015 | Can’t tell | Yes | Yes | Can’t tell | Yes |
| Basch, 2016 | Yes | Yes | Yes | Yes | Yes |
| Bruera, 2013 | Can’t tell | Yes | No | Yes | Yes |
| Chambers, 2017 | Yes | Yes | Yes | Yes | Yes |
| Cheung, 2017 | Yes | Can’t tell | No | Yes | Yes |
| Cheville, 2019 | Yes | Yes | Yes | Yes | Yes |
| Donovan, 2014 | Yes | Yes | Yes | Yes | Yes |
| Eldeib, 2018 | Yes | Yes | Yes | Yes | Yes |
| Flannery, 2018 | Yes | Can’t tell | No | Yes | No |
| Fox, 2019 | Yes | Yes | No | Yes | Yes |
| Gustafson, 2013 | Yes | Yes | No | Yes | Yes |
| Rose, 2009 | Can’t tell | Can’t tell | Yes | No | Can’t tell |
| Voruganti, 2017 | Yes | Yes | Yes | No | Yes |
| Yanez, 2015 | Can’t tell | Yes | Yes | Yes | Yes |
| Yount, 2014 | Yes | Yes | No | Yes | Yes |
| Bouchard, 2019 | Yes | Yes | Yes | Yes | Yes |

| Study | **MMAT – Quantitative non-randomised trials** | | | | |
| --- | --- | --- | --- | --- | --- |
|  | Are the participants representative of the target population? | Are measurements appropriate regarding both the outcome and intervention (or exposure)? | Are there complete outcome data? | Are the confounders accounted for in the design and analysis? | During the study period, is the intervention administered (or exposure occurred) as intended? |
| Alter, 1996 | Yes | Yes | Yes | No | Yes |
| Bensink, 2009 | Yes | Yes | No | No | Yes |
| Chow, 2001 | Yes | Yes | Yes | No | Yes |
| Cluver, 2005 | Yes | Yes | Yes | No | Yes |
| Dixon, 2010 | Yes | Yes | Yes | No | Yes |
| Fleisher, 2008 | Yes | Yes | Yes | No | Yes |
| Haddad, 2003 | Yes | Yes | No | No | Yes |
| Hennemann- Krause, 2015 | Yes | Yes | Yes | No | Yes |
| Keikes, 2019 | Yes | Yes | No | No | Yes |
| Liu, 2018 | Yes | Yes | Yes | No | Yes |
| Rasschaert, 2016 | Yes | Yes | Yes | No | Yes |
| Sardell, 2000 | Yes | Yes | Yes | No | Yes |
| Sherry, 2017 | Yes | Yes | Yes | No | Yes |
| Upton, 2016 | Yes | Yes | Yes | No | Yes |
| Watanabe, 2013 | Yes | Yes | No | No | Yes |
| Weaver, 2014 | Yes | Yes | Yes | No | Yes |
| Fox, 2019 | Yes | Yes | Yes | No | Yes |
| Schmitz, 2019 | Yes | Yes | Yes | No | Yes |
| Wright, 2018 | Yes | Yes | Yes | No | No |
| Chavarri-Guerra, 2020 | Yes | Yes | Yes | No | Yes |
| Friis, 2020 | Yes | Yes | Yes | No | No |
| Trojan, 2020 | Yes | Yes | Yes | No | Yes |
| Nemecek, 2019 | Yes | Yes | Yes | No | Yes |
